# Correspondence

**Published:** 1869-01

**Authors:** Edgar Park

**Affiliations:** Rec. Sec. A. D. Asso.


### ARTICLE IV.

St. Louis, Mo. December 22d, 1868.

*Editors of the American Journal of Dental Science.*

Gentlemen :—

By resolution of the American Dental Association convened in July last, the Secretary was instructed to cause to be published in the Dental Journals the following form of certificate to be presented to that body by delegates.

"This certifies that . . . . . . was duly appointed a delegate to the American Dental Association on the . . day of . . . . . . 18 . . by the Dental Society of . . . . . . and that said . . . . . . . is a dentist of good character and standing ; and at this time in regular practice."

Please therefore give this space in your Journal.

Respectfully

Edgar Park,

*Rec. Sec. A. D. Asso.*

---

